# Comparing the Efficacy of Midazolam Alone, With a Stress Ball, or With Hand-Holding and Conversation in Reducing Preoperative Anxiety in Elective Laparoscopic Cholecystectomy: A Randomised Controlled Trial

**DOI:** 10.7759/cureus.90900

**Published:** 2025-08-24

**Authors:** Usha Shukla, Jay Brijesh Singh Yadav, Shuchi Nigam, Mitul Mukherjee

**Affiliations:** 1 Anaesthesiology and Critical Care Medicine, Uttar Pradesh University of Medical Sciences, Saifai, Saifai, IND; 2 Anaesthesiology, Uttar Pradesh University of Medical Sciences, Saifai, Saifai, IND

**Keywords:** anxiety, laparoscopic cholecystectomy, midazolam, patient satisfaction, preoperative period

## Abstract

Introduction: Preoperative anxiety exists frequently and exerts an impact on overall surgical outcomes as well as the management of anaesthesia. The aim of this study was to evaluate the effectiveness of hand-holding and conversation with a stress ball, along with a control group of midazolam, in reducing preoperative anxiety.

Methods: We randomly divided 150 patients into three groups of 50 patients each: group A (hand-holding and conversation with midazolam), group B (stress ball use with midazolam), and group C (midazolam only). After taking informed and written consent, we recorded the Amsterdam Preoperative Anxiety and Information Scale (APAIS) score and haemodynamic parameters before and after intervention. We recorded the Visual Analogue Scale (VAS) score and patient satisfaction score in the postoperative period.

Results: The APAIS anxiety score was lowest in group A (6.2 ± 1.2) compared to group B (7.3 ± 1.3) and highest in group C (8.0 ± 1.1), respectively (P < 0.001). The minimum alveolar concentration (MAC) values for time points 15 and 30 minutes were 1.01 ± 0.06 and 1.03 ± 0.10 in group A, compared to 1.06 ± 0.10 and 1.07 ± 0.11 for group B and 1.07 ± 0.10 and 1.07 ± 0.09 for group C, respectively (P = 0.002 for 15 minutes). The patient satisfaction scores immediately after surgery were 4.2 ± 1.1 for group A, 3.7 ± 1.1 for group B, and 3.6 ± 0.9 for group C, respectively (P = 0.015).

Conclusion: Hand-holding and conversation with midazolam seems to be more effective in reducing preoperative anxiety compared to stress ball use in combination with midazolam and midazolam alone, and the hand-holding group had lower intraoperative anaesthetic requirement and higher patient satisfaction.

## Introduction

Tension, unease, or apprehension resulting from the expectation of danger, whether internal or external, is anxiety [1]. Anxiety also includes a collection of behavioural responses, typically categorised into state anxiety and trait anxiety. State anxiety is a transitory emotional condition [2]. Trait anxiety is a lifelong pattern of anxiety as a personality feature, which regularly hampers a person’s experiences [3]. Preoperative anxiety exists frequently and exerts an impact on overall surgical outcomes as well as the management of anaesthesia. Worldwide, the incidence of preoperative anxiety shows considerable variation, with estimates between 8% and above 80%. The correlation between preoperative anxiety and increased use of anaesthetic agents, delayed awakening, hemodynamic disturbances, postoperative discomfort, delayed wound healing, infection risk and prolonged hospital stays is widely acknowledged [4,5].

There are currently two different kinds of preoperative anxiety interventions: pharmacological and non-pharmacological. Sedatives and anti-anxiety medications are examples of pharmacological therapies [6]. The most widely used anxiolytics are midazolam, diazepam, ketamine and fentanyl, with midazolam the most popular [7].

Physical gestures such as the simple act of hand-holding can lower stress levels by having physiological effects and building a social connection [8]. Hand-holding may provide comfort during anxious moments, and incorporating a touch of conversation can distract the mind from worrying thoughts, making the whole pre-surgery experience easier to handle for patients suffering from excessive anxiety [9]. Non-pharmacological methods are often considered as an alternative to drug use due to the adverse effects of pharmacological treatments, which may include breathing difficulties, drowsiness, interaction with anaesthetic medicines and delayed recovery [10].

The purpose of our study was to reduce pharmacological drug therapy use and evaluate the efficacy of hand-holding with conversation and stress ball use in combination with midazolam and midazolam alone in relieving preoperative anxiety in adult patients undergoing elective laparoscopic cholecystectomy. The primary objective of this study was to determine the effect of hand-holding and conversation and stress ball use with midazolam on preoperative anxiety, using the Amsterdam Preoperative Anxiety and Information Scale (APAIS) [11]. The secondary objectives were to compare the intraoperative anaesthetic requirements based on depth of anaesthesia measured using the Bispectral Index (BIS) and compare haemodynamic parameters, postoperative pain using the Visual Analogue Scale (VAS) and the patient satisfaction score.

## Materials and methods

After receiving approval from the institutional ethical committee (Ethical Clearance Number: 62/2023-24), we conducted this prospective randomised controlled study in the Department of Anaesthesiology, Uttar Pradesh University of Medical Sciences, Saifai, India, from December 2023 to November 2024. We registered this study with the Clinical Trials Registry India (CTRI) (Reg. no. CTRI/2023/12/060560).

On the basis of a previous study conducted by Sriramka et al. [12], we determined the sample size using the formula:

$n = \frac{(\sigma_1^2 + \sigma_2^2)(Z_\alpha + Z_\beta)^2}{(\mu_1 - \mu_2)^2}$

After considering an alpha error of 0.05, a confidence interval of 95% and a power of 80%, we calculated the sample size to be a minimum of 47 in each group. Assuming a dropout rate of 5%, we took a sample size of 50 patients in each group. We conducted randomisation using a computer-generated random numbers table and conducted the group allocation through the sequentially numbered sealed opaque envelope technique. The envelopes were prepared in advance by an independent investigator who was not involved in patient recruitment or outcome assessment. Envelopes were opened sequentially only after enrolment, and monitoring staff periodically verified integrity and compliance with the allocation process. We recorded informed and written consent from all the patients after explaining the procedure and its related risks.

In our study, we included patients of either sex (male or female), the age group of 30-50 years, American Society of Anaesthesiologists (ASA) Physical Status (PS) class I and II and posted for the elective laparoscopic cholecystectomy. We excluded patients who did not give consent, had a previous history of anxiety disorders, neuropsychiatric disorders or chronic use of psychoactive medications, had visual and hearing impairment, and were taking beta blockers or had amputated upper limbs.

All the patients had undergone pre-anaesthetic check-up, including detailed history, general examination and clinical laboratory tests. On the day before surgery, we explained the study, the randomisation method and the technique of hand-holding and conversation, and we instructed all patients about how to use the stress ball.

**Figure 1:**
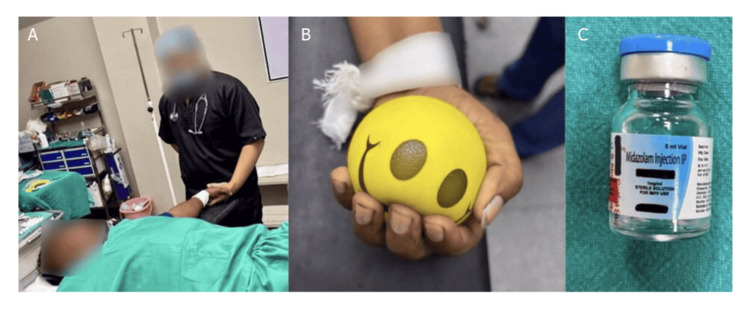
Non-pharmacological and pharmacological methods of decreasing anxiety (hand-holding and conversation (A), use of stress ball (B) and midazolam injection (C), respectively).

We informed patients how to rate the VAS for postoperative pain [13]. We instructed all patients to fast for at least eight hours for solid food and two hours for clear fluids prior to surgery. Premedication included oral administration of ranitidine 150 mg and alprazolam 0.25 mg on the night preceding the surgical procedure. We randomly divided 150 patients into three groups (Figure 1), with each group comprising 50 individuals.

Group A comprised injected patients with midazolam (0.05mg/kg IV) and conducted hand-holding and a conversation intervention for 15 minutes; Group B had injected patients with midazolam (0.05mg/kg IV) and gave them a stress ball to use for 15 minutes, squeezing it with every inspiration; Group C had injected patients with midazolam (0.05mg/kg IV) and instructed them to close their eyes for 15 minutes.

In the operating room, we attached the ASA standard monitoring devices (non-invasive blood pressure, ECG, pulse oximetry) and monitored and documented the baseline parameters, including the APAIS anxiety score and preoperative heart rate, systolic and diastolic blood pressure, mean arterial pressure (MAP) and peripheral oxygen saturation. We obtained intravenous access with an 18-G cannula and started Ringer's lactate infusion at 5-6 mL/kg.

We conducted an intervention with patients based on group allocation. Healthcare professionals of the same gender gave female and male patients a hand-holding intervention. The conversation was done by the same anaesthesiologist for all the patients. Topics of conversation included patient particulars, such as name, age, residence, profession, educational qualification, hospital stay experience and hobbies, briefing them about the anaesthetic procedure, assuring them about a safe and pain-free period of unconsciousness accompanied by an anaesthesiologist when they would be under anaesthesia, and other anaesthetic and procedure queries.

Anaesthesiologists not involved in the study recorded the APAIS anxiety score and haemodynamic parameters (heart rate and MAP). We attached four sensor electrodes of the Mindray BIS module (Mindray Medical International Ltd., Shenzhen, China) with BIS QUATRO sensors (Medtronic Inc., Dublin, Ireland) to the patients’ foreheads.

We pre-medicated all patients with fentanyl injection (2 μg/kg). We performed pre-oxygenation using 100% oxygen for a duration of three minutes. We induced patients with propofol injection (2-3 mg/kg or in a dose sufficient to cause loss of verbal command). We facilitated muscle relaxation by administering a loading dose of vecuronium at 0.1 mg/kg intravenously. After three minutes of ventilation, we conducted intubation (after achieving adequate relaxation) with a cuffed endotracheal tube of internal diameter 7.0 mm/7.5 mm in female patients and 8.0 mm/8.5 mm in male patients, respectively. We maintained anaesthesia with 33% oxygen, 66% nitrous oxide and isoflurane, maintaining BIS values between 40 and 60. We ensured adequate muscle relaxation by intermittent injections of vecuronium (0.02 mg/kg) every 20 minutes after a loading dose or when the patient showed signs of inadequate muscle relaxation. We administered paracetamol injection in a dose of 15 mg/kg IV (up to 1 gram) and tramadol injection in a dose of 2 mg/kg IV (up to 100mg) to maintain intraoperative analgesia. Around 30 minutes prior to skin closure, we administered an ondansetron injection (4 mg). At the end of surgery, we reversed muscle relaxation with neostigmine injection in a dose of 0.05 mg/kg IV and glycopyrrolate injection in a dose of 10 μg/kg IV. We extubated patients once all the criteria for extubating them were met. In the postoperative period, we maintained adequate analgesia by paracetamol injection in a dose of 15 mg/kg IV thrice a day (up to 1 gram/dose). We treated pain scoring ≥ 4 on the VAS [13] in the postoperative period by tramadol injection in a dose of 2 mg/kg IV (up to 100 mg/dose, maximum dosage 600 mg/day) and noted total opioid consumption used in 12 hours.

## Results

**Figure 2:**
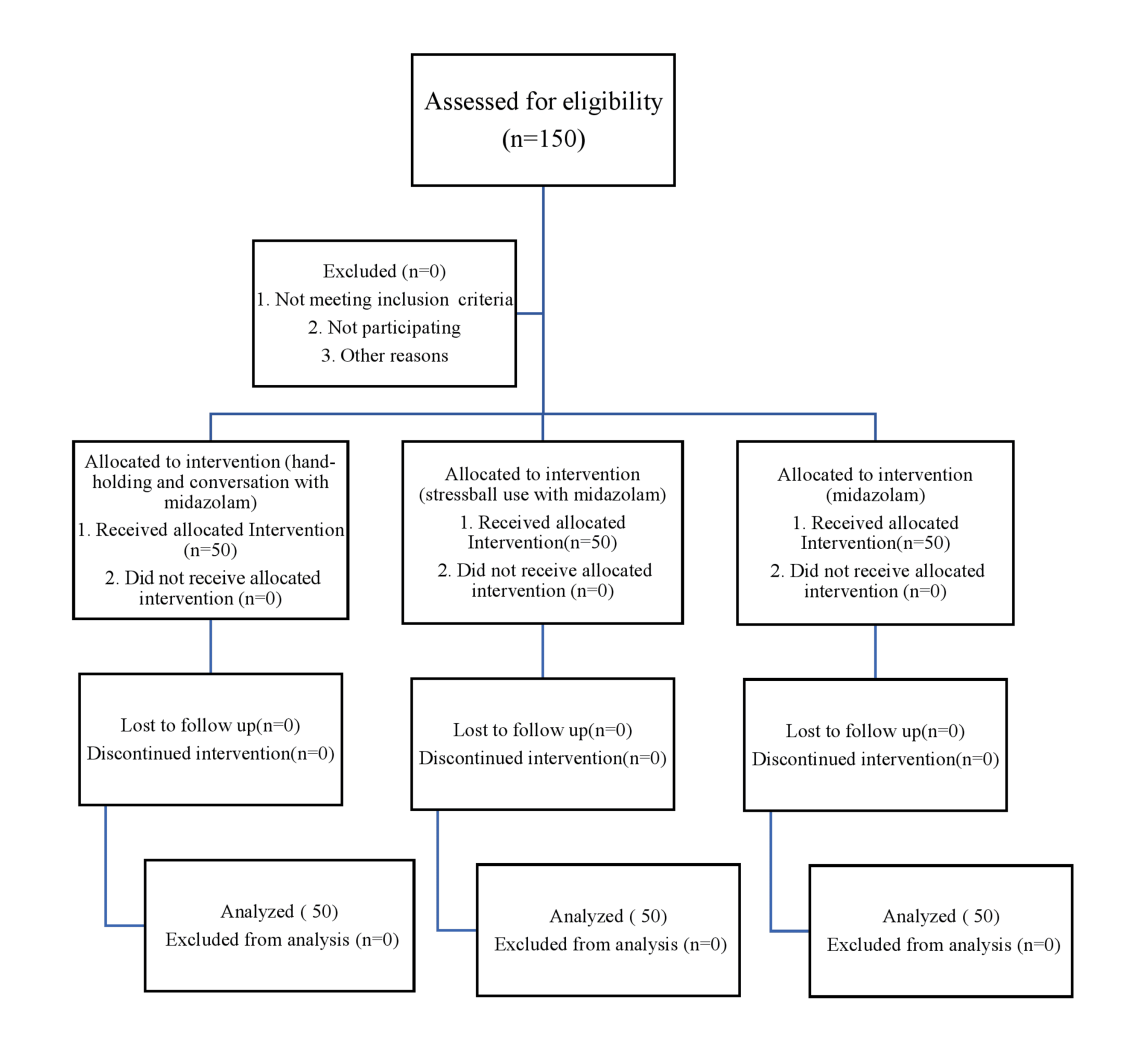
CONSORT flow diagram. CONSORT: Consolidated Standards of Reporting Trials

We recruited 150 patients for the study and excluded none, as shown in the Consolidated Standards of Reporting Trials (CONSORT) flow diagram (Figure 2). The demographic characteristics (age, gender, ASA PS Classification and BMI) of patients in groups A, B and C were comparable (P > 0.05) (Table 1). Before intervention, the APAIS anxiety score for the three groups (13.7 ± 1.8, 13.6 ± 1.7 and 14.1 ± 1.9) was comparable. After intervention in the preoperative period, group A had lower anxiety scores (6.2 ± 1.2) compared to group B (7.3 ± 1.3) and C (8.0 ± 1.1) (Table 2). During both intergroup and intragroup comparison, there was a statistically significant difference observed in pre-intervention and post-intervention anxiety scores in all the studied groups (P < 0.001).

**Table 1:** Comparison of demographic profile of patients among the studied groups. We analysed data expressed as mean and standard deviation by the unpaired t-test. We analysed data expressed in numbers by the chi-square test. We compared three groups using one-way analysis of variance (ANOVA). P < 0.05; statistically significant. n: number of patients in the group; BMI: body mass index; ASA PS: American Society of Anaesthesiologists Physical Status; M: male; F: female

Parameter	Group A (n = 50)	Group B (n = 50)	Group C (n = 50)	P-value
Age (years) (mean ± SD)	37.5 ± 6.4	38.8 ± 6.2	37.2 ± 6.7	0.400
Gender (M:F)	6:44	7:43	7:43	0.944
BMI (kg/m^2^) (mean ± SD)	24.8 ± 2.6	24.0 ± 2.6	24.7 ± 2.3	0.257
ASA PS (Class I/II)	42/8	40/10	41/9	0.873

**Table 2:** Comparison of APAIS anxiety scale scoring among the studies groups. We analysed data expressed as mean and standard deviation by the paired and unpaired t-test. We compared three groups using one-way analysis of variance (ANOVA). * P < 0.05; statistically significant. APAIS: Amsterdam Preoperative Anxiety and Information Scale; SD: standard deviation

APAIS Scale	Group A (Mean ± SD)	Group B (Mean ± SD)	Group C (Mean ± SD)	A vs. B	A vs. C	B vs. C	Overall (A vs. B vs. C)
				P-value	P-value	P-value	P-value
Before intervention	13.7 ± 1.8	13.6 ± 1.7	14.1 ± 1.9	0.656	0.366	0.173	0.379
After intervention	6.2 ± 1.2	7.3 ± 1.3	8.0 ± 1.1	<0.001*	<0.001*	0.005*	<0.001*
P-value^#^	<0.001*	<0.001*	<0.001*	^#^ Comparison of the mean value of paired observations			

**Figure 3:**
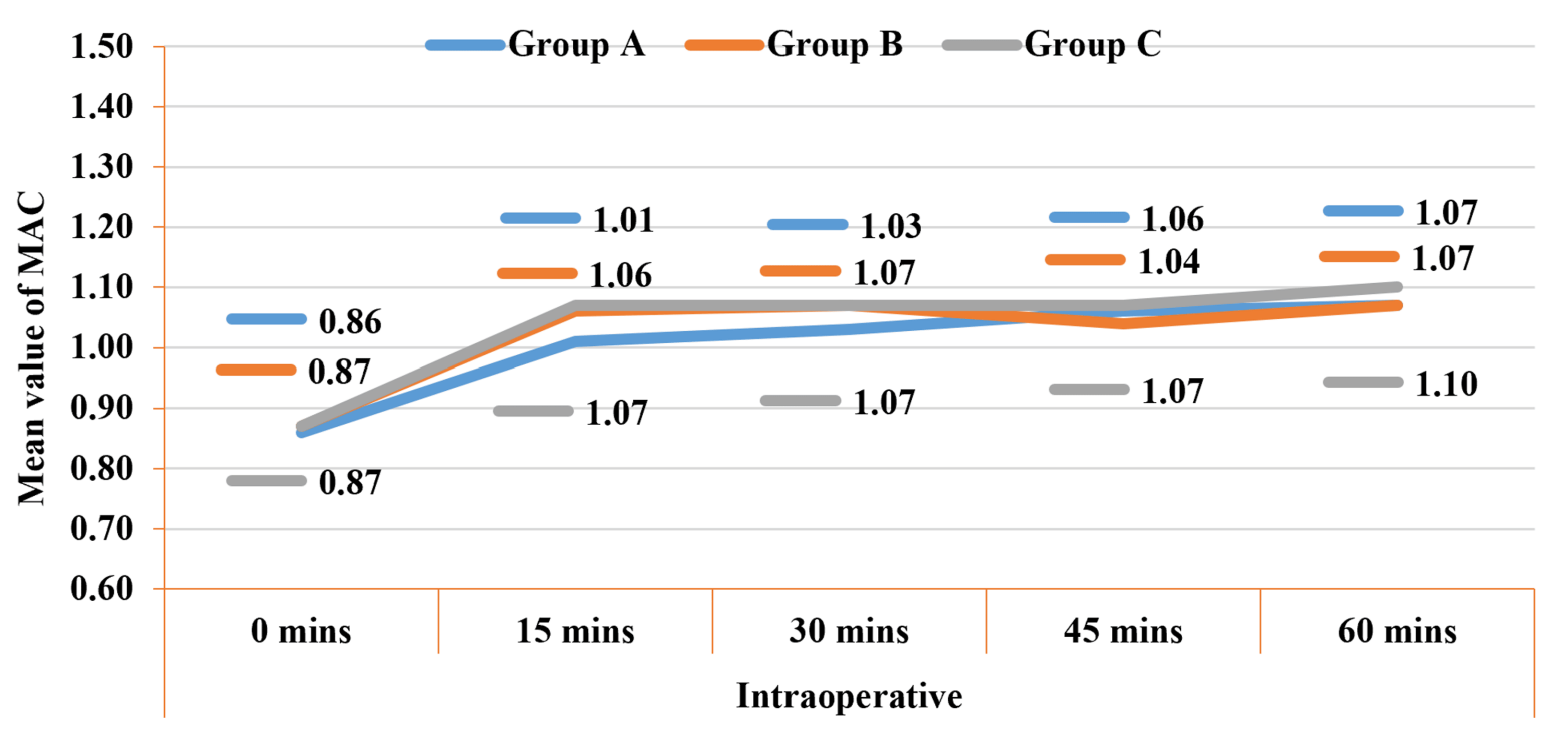
Comparison of MAC (intraoperative) among the studied groups. MAC: minimum alveolar concentration

Intraoperative anaesthetic requirements (minimum alveolar concentration, or MAC) were lowest at 15 and 30 minute time intervals in group A (1.01 ± 0.06 and 1.03 ± 0.10), followed by group B (1.06 ± 0.10 and 1.07 ± 0.11) and C (1.07 ± 0.10 and 1.07 ± 0.09) (Figure 3). During intergroup comparison, we found the difference in anaesthetic consumption to be statistically significant (P < 0.05).

Hemodynamic parameters (MAP) in the preoperative period before intervention in groups A, B and C (102.1 ± 4.6, 101.5 ± 4.9 and 102.8 ± 3.6) were comparable. Post-intervention, MAP was lowest in group A (93.9 ± 3.8), followed by groups B and C (96.1 ± 3.5 and 96.1 ± 3.7) (Table 3). We found the difference in mean values during intergroup comparison to be statistically significant between group A versus B (P = 0.004) and group A versus C (P = 0.003). During intragroup comparison of pre-intervention and post-intervention MAP values, we found the difference to be statistically significant (P < 0.001). Though there was not much difference in values of MAP among the groups but there is a wide difference in the pre-intervention and post-intervention MAP values.

**Table 3:** Comparison of mean values of MAP among the studied groups. We analysed data expressed as mean and standard deviation by the paired and unpaired t-test. We compared three groups using one-way analysis of variance (ANOVA). * P < 0.05; statistically significant. MAP: mean arterial pressure; SD: standard deviation

MAP	Group A (Mean ± SD)	Group B (Mean ± SD)	Group C (Mean ± SD)	A vs. B	A vs. C	B vs. C	Overall (A vs. B vs. C)
				P-value	P-value	P-value	P-value
Preoperative							
0 mins	102.1 ± 4.6	101.5 ± 4.9	102.8 ± 3.6	0.532	0.414	0.141	0.353
5 mins	98.2 ± 3.6	100.2 ± 4.8	100.4 ± 3.7	0.017*	0.003*	0.846	0.011*
10 mins	95.8 ± 2.5	98.0 ± 3.8	97.3 ± 3.2	0.001*	0.009*	0.343	0.003*
15 mins	93.9 ± 3.8	96.1 ± 3.5	96.1 ± 3.7	0.004*	0.003*	0.917	0.003*
P-value^#^	<0.001*	<0.001*	<0.001*	^#^ Comparison of the mean value of paired observations between 5 mins and 15 mins			
Intraoperative							
0 mins	96.0 ± 3.7	95.4 ± 3.0	96.1 ± 4.1	0.363	0.898	0.324	0.568
15 mins	93.4 ± 4.3	92.6 ± 2.7	92.3 ± 3.7	0.272	0.176	0.645	0.301
30 mins	93.0 ± 3.0	92.1 ± 3.1	92.1 ± 3.3	0.144	0.145	0.958	0.245
45 mins	92.4 ± 3.2	91.9 ± 2.4	92.6 ± 3.1	0.488	0.706	0.26	0.554
60 mins	92.6 ± 2.0	92.4 ± 1.0	92.1 ± 2.2	0.894	0.734	0.841	0.933
Postoperative							
Immediate	92.0 ± 2.8	91.3 ± 2.5	91.5 ± 2.7	0.185	0.339	0.719	0.386
3 hrs	91.8 ± 3.1	91.2 ± 2.8	91.0 ± 2.8	0.320	0.193	0.744	0.383
6 hrs	91.3 ± 3.2	90.5 ± 2.4	91.5 ± 2.6	0.162	0.709	0.047*	0.157
9 hrs	91.1 ± 2.7	90.4 ± 2.5	90.9 ± 2.7	0.169	0.614	0.389	0.386
12 hrs	90.9 ± 2.9	91.3 ± 2.3	90.7 ± 3.0	0.506	0.694	0.277	0.566

**Figure 4:**
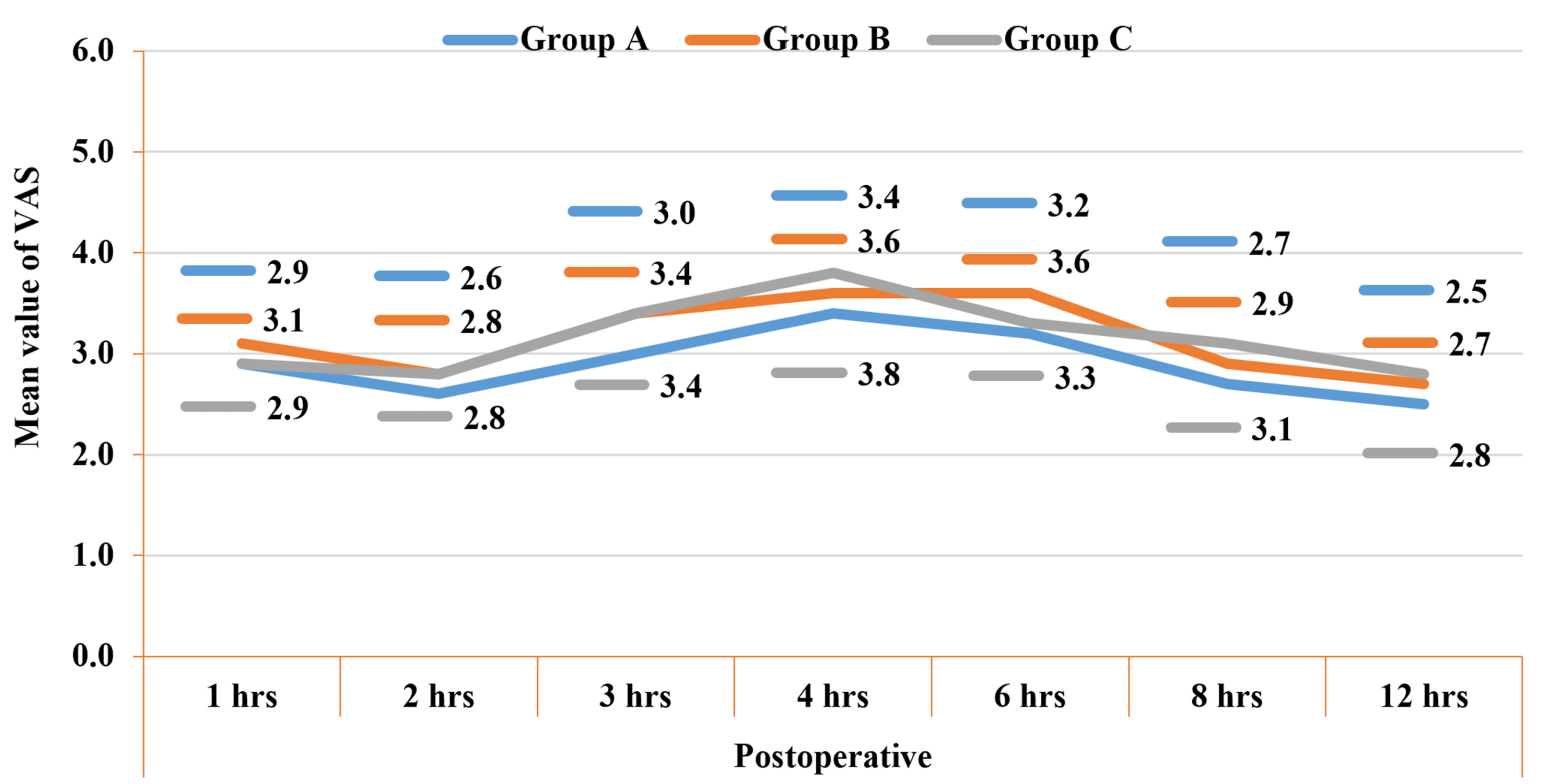
Comparison of VAS (postoperative) among the studied groups. VAS: Visual Analogue Scale

**Figure 5:**
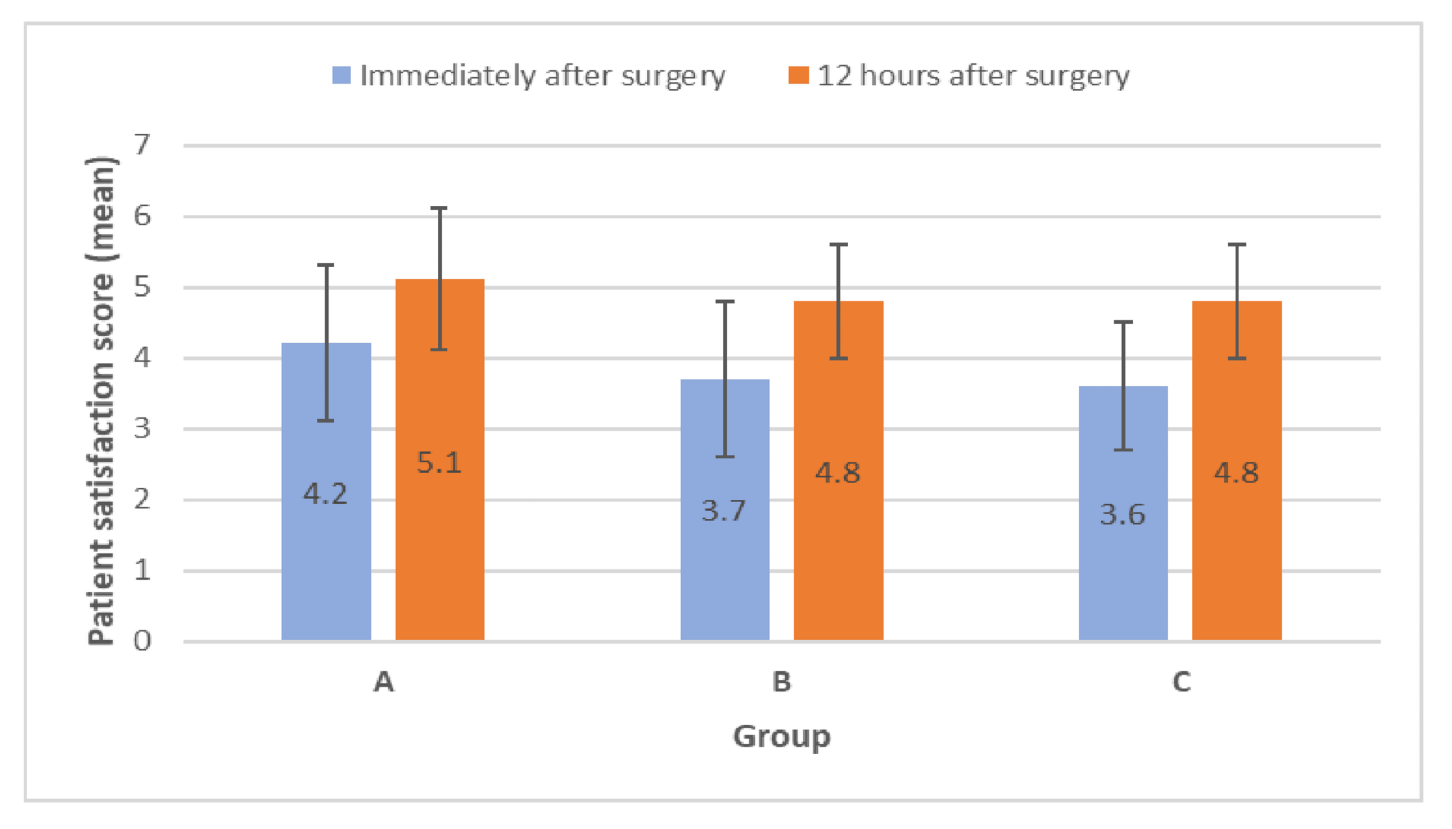
Comparison of patient satisfaction score among the studied groups.

In the postoperative period, during intergroup comparison, the mean values of the VAS score were statistically not significant among the studied groups (Figure 4). In the immediate postoperative period, group A patients reported higher satisfaction (4.2 ± 1.1) compared to group B (3.7 ± 1.1) and C (3.6 ± 0.9). However, patient satisfaction was comparable among the studied groups 12 hours after surgery (Figure 5).

## Discussion

We conducted this study with the aim of evaluating the effectiveness of hand-holding and conversation with a stress ball and midazolam in reducing preoperative anxiety in patients undergoing laparoscopic cholecystectomy.

Our study showed a statistically significant difference during intragroup comparison in pre-intervention and post-intervention APAIS scores (P < 0.001). This decrease in anxiety score may be due to hand-holding and conversation, which may lower stress levels by having physiological effects and building a social connection with the healthcare professional. Hand-holding may also provide comfort during anxious moments, and incorporating conversation can distract the mind from worrying thoughts. In 108 adult patients having lumbar spine procedures, Lal et al. [14] assessed the efficacy of hand-holding and discussion, both in conjunction with and without midazolam, in lowering preoperative anxiety. Their study reported greater change and lowest anxiety scores in hand-holding and conversation with the midazolam group (7.64 ± 1.05), followed by the hand-holding group (8.75 ± 1.21) and control group (11.64 ± 1.79) (P < 0.001). Sriramka et al. [12] made similar findings in studying the effect of hand-holding and conversation alone or with midazolam premedication on preoperative anxiety in 90 adult patients undergoing laparoscopic surgeries, with post-intervention APAIS scoring being lowest in hand-holding and conversation with the midazolam group (9.1 ± 4.7) in comparison to the hand-holding group (10.0 ± 3.8) and midazolam group (15.00 ± 3.6) (P < 0.001). These studies support the findings of our research.

Researchers believe that higher anxiety levels in the preoperative period increase the intraoperative anaesthetic requirements. In Maranets and Kain’s study [15] on 57 adult women undergoing bilateral laparoscopic tubal ligation, their findings were in agreement with our study. They reported lower intraoperative propofol requirements (110 ± 20 µg/kg/min) for maintenance of anaesthesia in the groups having lower anxiety scores in comparison to the medium and high anxiety groups (140 ± 40 µg/kg/min and 170 ± 70 µg/kg/min) (P = 0.03). Higher anaesthetic requirements in higher anxiety individuals may be because anxiety triggers the release of cortisol and adrenaline and increases the sensitivity to pain, requiring higher doses of anaesthetics to counteract the effects. Establishing a good rapport between the provider and patient is an important factor in alleviating anxiety and lowering intraoperative anaesthetic requirements during surgery.

Increased heart rate due to anxiety may lead to higher MAP. In Yadav et al.’s study [16] on 144 female patients admitted for caesarean section under regional anaesthesia and Sriramka et al. [12], they observed lower MAP in the group having lower anxiety levels. Post-intervention, Sriramka et al. [12] observed the lowest MAP values in the hand-holding and conversation group (77.0 ± 11.5 mm of Hg), followed by the hand-holding group and midazolam group, respectively (78.9 ± 12.4 mm of Hg and 80.8 ± 9.7 mm of Hg). The difference in mean MAP values was found to be statistically significant in both studies (P = 0.001 and P < 0.001, respectively). These observations reinforced our findings of having lower MAP after intervention in the groups having lower anxiety levels in the preoperative period.

Effectively managing anxiety in the preoperative period often leads to improved overall surgical and hospital stay experience and to better patient satisfaction scores. In our study, the hand-holding and conversation with midazolam intervention had higher satisfaction scores.

Yadav et al. [16] supported these findings. They reported a statistically significant difference in the patient satisfaction score during intragroup comparison of groups having higher and lower anxiety scores (P < 0.001). Yanes et al. [17], in assessing the effect of stress ball use and hand-holding on anxiety during skin cancer excision, reported similar findings of a correlation between lower anxiety levels and higher patient satisfaction scores.

## Conclusions

We conclude that hand-holding and conversation a better non-pharmacological interventions compared to a stress ball in lowering preoperative anxiety, which may lead to better intraoperative haemodynamic stability and improved overall patient satisfaction and may also decrease intraoperative anaesthetic consumption. Additional studies may seek to identify the best equilibrium among these interventions, investigating how well they perform in diverse groups of patients undergoing various surgical procedures.
